# Findings in magnetic resonance imaging for restaging locally advanced rectal cancer

**DOI:** 10.1007/s00384-024-04595-x

**Published:** 2024-01-30

**Authors:** Suvi Marjasuo, Laura Koskenvuo, Anna Lepistö

**Affiliations:** 1 Radiology, HUS Diagnostic Center, Helsinki University Hospital and University of Helsinki, Helsinki, Finland; 2 Tays Central Hospital, Imaging Services, PL 2000, 33521 Tampere, Finland; 3 Gastroenterological Surgery, Helsinki University Hospital, Helsinki, Finland; 4 Department of Surgery, Helsinki University Hospital and University of Helsinki, Helsinki, Finland; 5 Applied Tumor Genomics, Research Programs Unit Organization, University of Helsinki, Helsinki, Finland

**Keywords:** Rectal cancer, Locally advanced rectal cancer, MRI response

## Abstract

**Purpose:**

We aimed to assess the prognostic value of restaging magnetic resonance imaging (MRI) in rectal cancer after neoadjuvant therapy and compare long-course chemoradiotherapy (LC-CRT) to short-course radiotherapy with delayed surgery (SCRT-delay).

**Methods:**

This retrospective study included 267 patients with locally advanced rectal cancer (LARC) operated on between January 2016 and April 2019, all of whom received either LC-CRT or SCRT-delay in the neoadjuvant setting. The primary outcomes were overall survival (OS) and cancer-specific survival (CSS) based on radiological response assessed using the magnetic resonance tumor regression grade (mrTRG).

**Results:**

In the LC-CRT group, cumulative 1-, 3-, and 5-year OS rates were 94.8%, 86.4%, and 79.0%, while in the SCRT-delay group, they were 83.3%, 68.9%, and 68.9% (*P* = 0.017). For CSS in the LC-CRT group, cumulative rates were 96.9%, 90.3%, and 85.0%, and in the SCRT-delay group, they were 88.6%, 81.4%, and 81.4% (*P* = 0.222). There were no significant differences in total histological response rates or local recurrence rates between the treatment groups. The good and moderate response group (mrTRG 1–3) had significantly better cumulative 1-, 3-, and 5-year OS and CSS compared to the poorer response group (mrTRG 4–5) (*P* = 0.023 for OS and *P* = 0.048 for CSS).

**Conclusion:**

Unfavorable MRI response is a sign of poor prognosis in LARC. SCRT-delay is comparable to LC-CRT concerning the oncological outcome.

## Introduction

Globally, colorectal cancer affects 1–2 million people each year, with 600,000 of them succumbing to the disease. Roughly one-third of all colorectal cancer cases involve the rectum [1]. The choice of treatment for rectal cancer primarily depends on the stage of the cancer, although other factors may also be considered. The stage of the disease is determined by radiological examinations. Disseminated cancers are usually treated with systemic chemotherapy, while oligometastatic and locally limited cancers may undergo surgery following neoadjuvant therapy [2].

Optimized pelvic magnetic resonance imaging (MRI) provides high-quality imaging of the rectum and its surrounding structures, offering detailed anatomical information and some functional insights through diffusion-weighted imaging. In initial staging, MRI is utilized for locoregional clinical staging, identification of poor prognostic factors such as extramural venous invasion (EMVI), and determination of preoperative management and surgical extent [3]. A staging computed tomography (CT) scan of the thorax and abdomen is conducted to detect potential metastases and to assist in assessing the functional status of the bowel [2]. In the restaging setting, rectal MRI proves valuable in evaluating tumor regression and tailoring surgical planning [4].

Patients with locally advanced rectal cancer (LARC) require neoadjuvant therapy to enhance the likelihood of curative tumor resection and reduce the risk of recurrence. The standard neoadjuvant therapy regimen is long-course chemoradiotherapy (LC-CRT). A shorter treatment regimen without chemotherapy is more suitable for elderly and frail patients, as well as those with contraindications for chemotherapy. Comparable short- and long-term outcomes to conventional LC-CRT have been achieved with an alternative treatment approach of short-course radiotherapy followed by delayed surgery (SCRT-delay) [5, 6], but disease-free survival has been reported to be better after LC-CRT [7].

Prior studies have established that performing MRI in the post-neoadjuvant setting yields precise information about circumferential resection margin and response to (chemo)radiotherapy [8–10]. Consequently, it has become a pivotal tool in strategizing surgical approaches. According to a prospective study, the response to neoadjuvant treatment for LARC, as assessed by MRI, seems to serve as a predictive marker for varying survival outcomes based on the response [3]. Due to partly contradictory data on oncological outcomes after LC-CRT and SCRT-delay, we wanted to study the outcome in our large tertiary rectal cancer center, providing substantial number of patients having homogenous preoperative multidisciplinary team evaluation.

The objective of this study was to assess the prognostic significance of the response to neoadjuvant treatment assessed with MRI in predicting survival after neoadjuvant treatment for rectal cancer. Additionally, we compared the outcomes between LC-CRT and SCRT-delay.

## Methods

### Patient characteristics

**Fig. 1:**
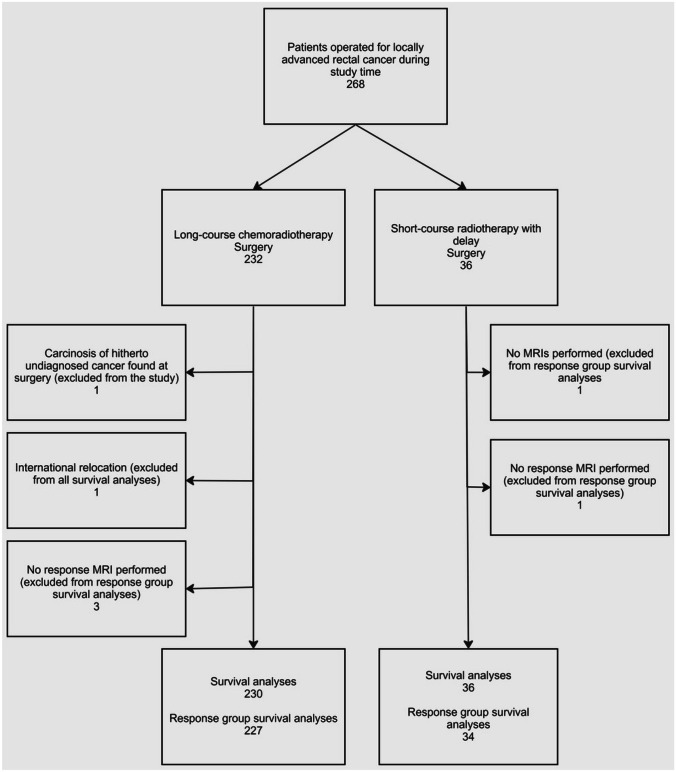
Patient flowchart

In this retrospective, population-based study, we included all previously untreated patients (*n* = 268) with LARC, who underwent surgery after LC-CRT or SCRT-delay in the neoadjuvant setting between January 2016 and April 2019 at Helsinki University Hospital. Participation of patients is shown in the flowchart (Fig. 1). Seven patients were excluded from the study because of contraindications for MRI, lack of MRI, international relocation, and carcinosis of another, hitherto undiagnosed cancer detected at surgery.

Data were collected from medical records and encompassed various information, including gender, age at diagnosis, histological type, tumor location as determined by colonoscopy and MRI, radiological assessment of metastatic lymph nodes and distant metastases, details of neoadjuvant treatment, response assessment using MRI, the duration between neoadjuvant therapy and surgery, type of surgery performed, locations of cancer recurrence, overall survival, and causes of death.

LARC was defined as rectal cancer with a threatened margin or invading adjacent tissue/organ or with lateral para-iliacal lymph nodes [2], and neoadjuvant LC-CRT or SRCT-delay was administered to the patients following national guidelines. LC-CRT was the preferred treatment method for the majority, whereas SCRT-delay was selected for frail, elderly patients considered unsuitable for chemotherapy. Patients underwent CT scans of the chest, abdomen, and pelvis, as well as rectal MRI, for initial staging and subsequent restaging after neoadjuvant therapy. Tumor histology was confirmed through preoperative endoscopic biopsies. The treatment decisions of all patients were made in multidisciplinary team meetings, which included a colorectal surgeon, an oncologist, an abdominal radiologist, and a pathologist.

LC-CRT consists of multiple radiation sessions (28 × 1.8 Gray (Gy) = 50.4 Gy to the tumor site, 45 Gy of which also to the lymph nodes) combined with capecitabine. The SCRT-delay regimen consists of five radiotherapy doses, 5 Gy each, on consecutive days without any chemotherapy. The surgery was delayed following LC-CRT or SCRT-delay, typically with a planned delay of 6–8 weeks. Adjuvant chemotherapy after surgery was considered if metastatic lymph nodes, lymphovascular or neural invasion, histological grade 3, or tumor budding grade 3 were detected in the surgical specimen.

Pathologic assessment was made for all surgical specimens according to national guidelines. The Dworak grading system is defined as follows: 1, no regression; 2, dominant tumor mass with obvious fibrosis and/or vasculopathy; 3, dominant fibrotic changes with few tumor cells or groups; 4, very few tumor cells in fibrotic tissue with or without mucous substance; and 5, no tumor cells and only a fibrotic mass.

Data collection from patient records occurred at least 2 years after surgery. The follow-up period was defined as the duration in months from the date of surgery until the last contact with the healthcare providers or death.

The study design and reporting adhere to the STROBE (Strengthening the Reporting of Observational Studies in Epidemiology) guidelines, as recommended by the EQUATOR Network.

This study was approved by the Research Administration of Helsinki University Hospital (HUS 40/2021). As this was a retrospective registry study with no patient intervention, ethics committee approval and informed consent were not required by the Finnish National Legislation.

### Radiological technique

CT images were obtained with contrast medium in the portal venous phase in all but two patients. Contrast medium was omitted in two patients for technical/patient-related reasons. A multi-slice helical CT scanner was used in all patients. The images were interpreted by experienced radiologists.

MR (magnetic resonance) images of the rectum were obtained with Siemens Magnetom Skyra 3.0 T or Siemens Magnetom Avanto 1.5 T scanners using T2-weighted imaging in three planes as well as diffusion-weighted imaging in the oblique plane perpendicular to the lumen of the bowel wall comprising the tumor. One patient had an absolute contraindication to MR imaging. In four patients, no restaging MRI was performed for unknown reasons. No filling of the rectum was used, but the patients received a small enema before the scan to minimize susceptibility artifacts. The tumor regression after neoadjuvant therapy was described using a five-grade magnetic resonance tumor regression grade (mrTRG) [5]. MrTRG was defined as follows: 1, completely normalized rectal wall; 2, no obvious residual tumor, dense fibrosis signifying minimal residual disease or no tumor; 3, 50% fibrosis or mucin, and visible intermediate signal; 4, little areas of fibrosis or mucin but mostly tumor; and 5, intermediate signal intensity, same appearances as the original tumor. Favorable response was defined as mrTRG 1–3 (less than 50% viable tumor left) and unfavorable response as mrTRG 4–5 (more than 50% viable tumor left). The total radiological response was defined as mrTRG 1–2. The MR images were interpreted by experienced abdominal radiologists and reported using a structured report.

### Statistical analysis

Continuous variables were reported as medians and ranges. Categorical variables were compared using the Chi-square test, or Fisher’s exact test if expected cases in one cell were fewer than five. Time to recurrence was measured from the date of surgery. Cumulative overall survival (OS), cancer-specific survival (CSS), and recurrence distributions were analyzed by the Kaplan–Meier method and were compared using the log-rank test. Confounding variables were adjusted for using the Cox regression analysis. The multivariate Cox regression analysis included variables that exhibited a significance level of *P* < 0.10 in univariable analyses. Statistical analyses were performed using IBM SPSS Statistics software version 25 (IBM Corporation, Armonk, NY, USA). *P* < 0.05 was considered statistically significant. Missing values were excluded from the analyses of that particular variable.

## Results

### Patient characteristics

Of the 261 patients included in the study, 227 (87.0%) received LC-CRT, and 34 (13.0%) received SCRT-delay. The median follow-up time was 39 (range 24–64) months.

Synchronous metastases were identified in 26 patients (10.0%) (grade IV). The metastatic sites included the liver (nine patients), lymph nodes in the para-aortic (three patients), external iliac (four patients), groin regions (two patients), lungs (seven patients), and a combination of both the liver and lungs in one patient. Among the patients with grade IV disease, 22 patients underwent LC-CRT, and four received SCRT-delay. In line with the overall cohort, SCRT-delay was selected for those patients diagnosed with stage IV disease who were considered too frail to undergo LC-CRT. Additionally, 53 patients (20.3%) experienced metachronous metastases during the follow-up period.

The median delay between the termination of neoadjuvant therapy and surgery was 7 (range 4–31) weeks. Six patients underwent hepatic resection due to metastases during the delay time. One patient underwent nephrectomy due to a malignant renal tumor during the delay period.

The predominant procedure performed was anterior rectal resection, which accounted for 134 (50.2%) cases (Table 1). Total or partial mesorectal excision (TME/PME) was employed for all patients. In cases where the surgical margin was at risk, additional tissue outside the mesorectal fascia was removed to ensure a clear margin free from the tumor. Multivisceral resection was necessary in 49 cases (18.4%).

A total of 199 (74.8%) patients received adjuvant chemotherapy.

**Table 1:** Patient characteristics

	Chemoradiotherapy (*n* = 227)	Short-course radiotherapy with delayed surgery (*n* = 34)	P*
Sex, male	141 (62.1%)	18 (52.9%)	0.513
Age (mean ± standard deviation), years	64.5 ± 11.1	79.1 ± 7.8	< 0.001*
Visceral metastasis at the time of diagnosis	22 (9.7%)	4 (11.8%)	0.758
Type of surgery			0.117
Anterior resection	122 (53.7%)	9 (26.5%)	
Abdominoperineal excision	31 (13.7%)	7 (20.6%)	
Extended abdominoperineal excision	65 (28.7%)	17 (50.0%)	
Hartman procedure	7 (3.1%)	1 (2.9%)	
Proctocolectomy	1 (0.4%)	0	
Palliative colostoma	1 (0.4%)	0	
Macroscopically curative surgery	216 (95.2%)	34 (100%)	0.667
Postoperative chemotherapy			< 0.001*
Yes	190 (83.7%)	6 (17.6%)	
Missing data	6 (2.6%)	4 (11.8%)	
Mortality during follow-up			
Overall	37 (16.3%)	9 (26.5%)	0.152
Cancer-related	26 (11.5%)	6 (17.6%)	0.397

*P-values computed using the chi-square test

The LC-CRT- and SCRT-delay groups differed according to age, with patients being older in the SCRT-group, as well as according to postoperative chemotherapy, with the CRT-group receiving adjuvant treatment more often (Table 1).

### Disease characteristics

Out of the total patient cohort, rT4 was the most common classification with 163 individuals (62.5%) (Table 2). Post-neoadjuvant rT category was higher in the SCRT-delay group than in the LC-CRT group (Table 2). Also, the histological T category was higher in the SCRT-delay group, and better histological tumor regression grade was achieved more often in LC-CRT than in the SCRT-delay group (Table 3).

**Table 2:** Radiological characteristics

	Chemoradiotherapy (*n* = 227)	Short-course radiotherapy with delayed surgery (*n* = 34)	P*
Radiological T category at the time of diagnosis			0.120
T2	3 (1.3%)	1 (2.9%)	
T3 MRF −	27 (11.9%)	1 (2.9%)	
T3 MRF +	60 (26.4%)	8 (23.6%)	
T4a	47 (20.8%)	1 (2.9%)	
T4b	90 (39.6%)	23 (67.7%)	
Radiological N category at the time of diagnosis			0.080
N0	67 (29.5%)	14 (41.2%)	
N1	76 (33.5%)	14 (41.2%)	
N2	84 (37.0%)	6 (17.6%)	
Extramural venous invasion detected with magnetic resonance imaging at the time of diagnosis	100 (44.1%)	9 (26.5%)	0.063
Postneoadjuvant radiological T category			0.011*
Tx	37 (16.3%)	0	
T2	15 (6.6%)	3 (8.8%)	
T3 MRF −	45 (19.8%)	3 (8.8%)	
T3 MRF +	29 (12.8%)	9 (26.5%)	
T4a	40 (17.6%)	2 (5.9%)	
T4b	61 (26.9%)	17 (50.0%)	
MRI tumor regression grade			0.053
Grade 1 (complete radiological response)	0	0	
Grade 2 (good response)	44 (19.4%)	2 (5.9%)	
Grade 3 (moderate response)	132 (58.1%)	18 (52.9%)	
Grade 4 (slight response)	46 (20.3%)	12 (35.3%)	
Grade 5 (no response)	5 (2.2%)	2 (5.9%)	
Postneoadjuvant radiological N category			0.563
N0	140 (61.7%)	18 (52.9%)	
N1	66 (29.1%)	13 (38.3%)	
N2	21 (9.2%)	3 (8.8%)	
Postneoadjuvant radiological extramural venous invasion	68 (30.0%)	8 (23.5%)	0.546

*MRF* mesorectal fascia, *MRI* magnetic resonance imaging

*P-values computed using the chi-square test

**Table 3:** Histopathological characteristics of the surgical specimens

	Chemoradiotherapy (*n* = 227)	Short-course radiotherapy with delayed surgery (*n* = 34)	P*
Histopathological diagnosis			0.175
Invasive adenocarcinoma	182 (80.2%)	27 (79.5%)	
Invasive mucinous adenocarcinoma	16 (7.0%)	3 (8.8%)	
Mixed adenoneuroendocrine carcinoma	0	1 (2.9%)	
Adenosquamous carcinoma	1 (0.4%)	0	
Adenoma, high-grade dysplasia	2 (0.9%)	1 (2.9%)	
Fibrosis	26 (11.5%)	2 (5.9%)	
Histological tumor grade			0.613
1	25 (11.0%)	5 (14.7%)	
2	151 (66.5%)	24 (70.6%)	
3	15 (6.6%)	1 (2.9%)	
Missing data	36 (15.9%)	4 (11.8%)	
Histological T stage			0.048*
T0	29 (12.8%)	5 (14.7%)	
T1	10 (4.4%)	2 (5.9%)	
T2	48 (21.1%)	6 (17.6%)	
T3	107 (47.2%)	12 (35.3%)	
T4	32 (14.1%)	9 (26.5%)	
Missing data	1 (0.4%)	0	
Histological N stage			0.426
N0	147 (64.8%)	23 (67.7%)	
N1a	21 (9.3%)	2 (5.9%)	
N1b	25 (11.0%)	4 (11.8%)	
N1c	5 (2.2%)	1 (2.9%)	
N2a	12 (5.3%)	3 (8.8%)	
N2b	15 (6.6%)	0	
Nx	1 (0.4%)	1 (2.9%)	
Missing data	1 (0.4%)	0	
Lymphovascular or perineural invasion			0.759
Lymphovascular	22 (9.7%)	5 (14.7%)	
Perineural	26 (11.5%)	4 (11.8%)	
Both	1 (0.4%)	0	
Missing data	1 (0.4%)	0	
Dworak tumor regression grade			0.020*
0–1 (none or minimal response)	39 (17.2%)	13 (38.2%)	
2 (moderate response)	105 (46.2%)	13 (38.2%)	
3 (near-complete response)	51 (22.5%)	3 (8.8%)	
4 (complete response)	28 (12.3%)	4 (11.9%)	
Missing data	4 (1.8%)	1 (2.9%)	

*P-values computed using the chi-square test

During the course of follow-up, 12 patients (4.6%) developed local recurrence. Three recurrences were located in the anastomotic area, three in the lateral wall of the pelvis, five in the presacral region, and one adjacent to the pelvic floor reconstruction material.

### Survival rates and local recurrences in different treatment groups

In all patients the cumulative overall survival (OS) rates at 1, 3, and 5 years were 92.9%, 84.1%, and 77.4%, respectively. The cumulative OS rates at 1, 3, and 5 years were 94.8%, 86.4%, and 79.0% in the LC-CRT group and 83.3%, 68.9%, and 68.9% in the SCRT-delay group, respectively (*P* = 0.017).

The CSS rates in the entire study group at 1, 3, and 5 years were 95.8%, 89.2%, and 84.3%, respectively. In the LC-CRT group, the cumulative CSS rates at 1, 3, and 5 years were 96.9%, 90.3%, and 85.0%, while in the SCRT-delay group, they were 88.6%, 81.4%, and 81.4%, respectively (*P* = 0.222).

A total of 11 patients (4.8%) in the LC-CRT group experienced local recurrence, while one patient (2.9%) in the SCRT-delay group had local recurrence (*P* = 1.000). The median time from surgery until local recurrence was 11 (range 3–36) months.

### Radiological assessment of response to neoadjuvant therapy

Altogether, 195 (74.7%) patients had mrTRG 1–3 and 66 (25.3%) patients had mrTRG 4–5. The cumulative OS at 1-, 3-, and 5 years for the mrTRG 1–3 patients was 95.4%, 88.6%, and 79.9%, and for the mrTRG 4–5 patients, 86.2%, 72.5%, and 72.5%, respectively (*P* = 0.023).

In a univariate Cox regression analysis, poor response to neoadjuvant (chemo)radiotherapy (mrTRG 4–5) was a risk factor for poor survival, as were also PNI or LVI detected in the surgical specimens, EMVI at baseline MRI, and pN positivity. In a multivariate regression analysis, only CRM < 1 mm was associated with poor survival. (Table 4).

**Table 4:** Univariate and multivariate Cox regression analyses of variables for association with overall mortality (*n* = 261)

	**Univariate**		**Multivariate**	
	**HR (95% CI)**	***P***	**HR (95% CI)**	***P***
CRM < 1 mm	4.26 (2.26–8.02)	< 0.001*	2.89 (1.48–5.65)	0.002*
PNI or LVI detected in the surgical specimen	3.25 (1.82–5.81)	< 0.001*	1.83 (0.92–3.63)	0.086
EMVI diagnosed with baseline MRI	1.79 (0.99–3.20)	0.051	1.19 (0.64–2.19)	0.583
Metastatic lymph nodes detected in the surgical specimen	2.89 (1.61–5.20)	< 0.001*	1.75 (0.89–3.42)	0.105
mrTRG 4–5 vs 1–3	1.98 (1.09–3.60)	0.026*	1.53 (0.83–2.81)	0.175

*HR* hazard ratio, *CI* confidence interval, *CRM* circumferential resection margin, *PNI* perineural invasion, *LVI* lymphovascular invasion, *EMVI* extramural venous invasion, *MRI* magnetic resonance imaging, *mrTRG* MRI tumor regression grade

**Fig. 2:**
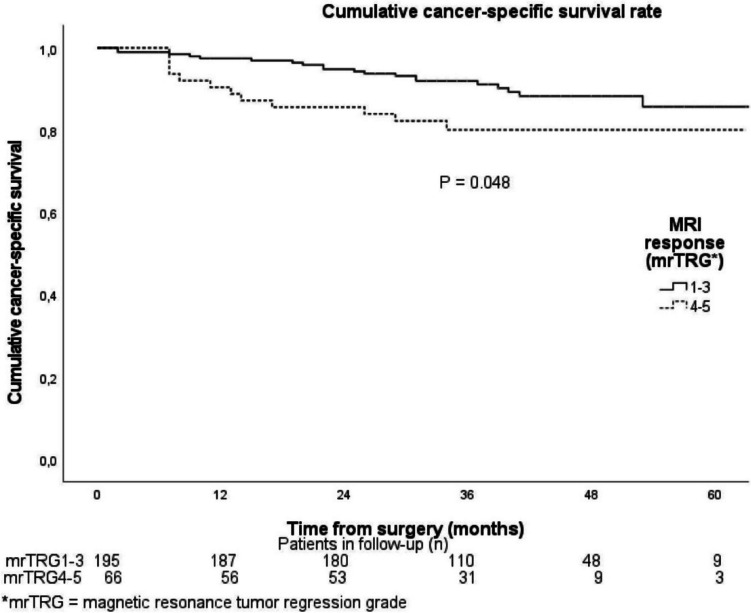
Cumulative risk of cancer-specific death between the favorable and unfavorable magnetic resonance imaging response groups in the Kaplan–Meier analysis

The cumulative CSS at 1, 3, and 5 years for the mrTRG 1–3 patients was 97.4%, 92.0%, and 85.7%, and for the mrTRG 4–5 patients, 90.4%, 80.1%, and 80.1%, respectively. (*P* = 0.048) (Fig. 2).

The Cox regression analysis of different risk factors for CSS is shown in Table 5.

**Table 5:** Univariate and multivariate Cox regression analyses of variables for association with cancer-specific mortality (*n* = 261)

	**Univariate**		**Multivariate**	
	**HR (95% CI)**	***P***	**HR (95% CI)**	***P***
CRM < 1 mm	5.87 (2.86–12.06)	< 0.001*	3.38 (1.58–7.23)	0.002*
PNI or LVI detected in the surgical specimen	5.66 (2.79–11.47)	< 0.001*	2.80 (1.22–6.45)	0.015*
EMVI diagnosed with baseline MRI	2.38 (1.17–4.84)	0.016*	1.32 (0.62–2.78)	0.474
Metastatic lymph nodes detected in the surgical specimen	4.47 (2.12–9.45)	< 0.001*	2.09 (0.88–4.95)	0.095
mrTRG 4–5 vs 1–3	2.02 (0.99–4.14)	0.054	1.42 (0.68–2.94)	0.348

Cumulative risk of cancer-specific death between the favorable and unfavorable magnetic resonance imaging response groups in Kaplan–Meier analysis. *HR* hazard ratio, *CI* confidence interval, *CRM* circumferential resection margin, *PNI* perineural invasion, *LVI* lymphovascular invasion, *EMVI* extramural venous invasion, *MRI* magnetic resonance imaging, *mrTRG* MRI tumor regression grade

The mrTRG 1–3 patients had a total of eight local recurrence cases, and the local recurrence rate at 5 years was 7.5%, while there was a total of four cases of local recurrence in the mrTRG 4–5 patients, and the corresponding rate was 8.0%, respectively (*P* = 0.312). The median time to local recurrence in patients categorized as mrTRG 1–3 was 12 months, and in patients with mrTRG 4–5, it was 8.5 months (*P* = 0.490).

MrTRG 4–5 was equally common in patients with and without synchronous metastases (5 (19.2%) vs 60 (25.5%), *P* = 0.490).

### Radiological assessment compared with histopathological assessment

MrTRG 1–2 was observed in 46 (17.2%) patients. In a pathological examination in 32 (11.9%) cases, the Dworak grade was 5. Of the patients with radiological assessments mrTRG 1–2, 16 (35.6%) appeared as total responders (Dworak 5) in the histopathologic examination. Conversely, 16 (50.0%) patients of those with the histopathological total response (Dworak 5 grade) were radiologically assessed as mrTRG 1–2 responders. The rate of the histopathological total response was similar in patients in the LC-CRT and SCRT-delay groups (12.4% vs 11.4%, *P* = 1.000).

## Discussion

In this large retrospective study, we found that patients who underwent LC-CRT had better OS compared to those who received SCRT-delay. Furthermore, we discovered that patients treated for LARC who exhibited a favorable response on MRI following neoadjuvant therapy had improved cumulative OS and CSS compared to patients with a poor response. However, it is important to note that this favorable response did not lead to a reduction in local recurrence rates. The lower survival rates observed in patients with a minimal MRI response indicate that this may be indicative of a more aggressive form of the disease. After accounting for confounding factors including a threatened surgical margin, lymphovascular or perineural invasion, EMVI diagnosed at the baseline MRI, and metastatic lymph nodes, a poorer response did not manifest as an independent risk factor for adverse OS or CSS in multivariate regression analysis of our study. This also suggests that poor response is a sign of biologically aggressive disease containing above mentioned biological risk factors. In part, this observation may also be attributed to the relatively small size of the poorer response group (*n* = 66, 25.3%), because only CRM < 1 mm remained as an independent risk factor for poor OS in multivariate analysis. The median time until local recurrence was similar between groups with different mrTRG.

While patients who underwent LC-CRT demonstrated better OS in comparison to those who received SCRT-delay, we did not observe any significant differences in CSS or local recurrence rates. The difference in OS between the two treatment groups can be attributed to patient selection. In our study, the patients in the SCRT-delay group tended to be older. Older and more fragile patients are typically ineligible for LC-CRT and are often at a higher risk of mortality due to other underlying health conditions.

In terms of other oncological outcomes, our results are in line with some previously published studies [11, 12]. The oncological outcome was not inferior in the SCRT-delay group, even though histological tumor regression grade was poorer and postoperative chemotherapy was given more sparsely. One randomized controlled trial [7] indicated that LC-CRT improved disease-free survival at the 3-year mark when compared to SCRT-delay, without a significant impact on OS. Considering all these factors, it appears that SCRT-delay is not inferior to LC-CRT when administered in the neoadjuvant setting for LARC.

Previous research has indicated that histopathological complete response occurs in approximately 10–30% of LARC cases treated with neoadjuvant LC-CRT [13–17] and around 10% of LARC cases treated with neoadjuvant SCRT-delay [18]. In our study, the overall rate of pathological complete response among all patients was 11.9%, and no statistically significant difference was observed between the two neoadjuvant therapy groups, although as a whole, better histological tumor regression was observed in the LC-CRT group. Interestingly, we found that MRI was not reliable in accurately identifying total histopathological responders. Only 35.6% of those with a radiologically good response (mrTRG 2) were histopathological total responders, and only 50% of those classified as histopathological total responders exhibited complete fibrosis on MRI. In a prior study [19], it was reported that MRI successfully identified total responders in 84.4% of cases. However, it is worth noting that in this study, the authors defined mrTRG 1–3 as a radiologically good response. As a result, their false-positive rate was considerably higher, with 84.6% of those classified as mrTRG 1–3 not actually being total responders.

When used in restaging, MRI seemed to be reliable in guiding surgical planning, as the local recurrence rate was not increased even when the radiological response was minor. Even in such cases, the radiologist plays a pivotal role in the preoperative restaging by providing the surgeon with a guide for planes of excision [20]. An unfavorable MRI response in the post-neoadjuvant setting could be an independent prognostic risk factor for LARC patients. Further research is necessary to understand the biological mechanisms behind more aggressive diseases and explore ways to develop more personalized treatment regimens based on these mechanisms [21]. Recent research efforts have focused on investigating the addition of neoadjuvant chemotherapy to the standard therapy regimen yielding promising results [22, 23].

This study had some limitations. The interpretation of our findings is somewhat constrained by the retrospective design of our study, which may impede the identification of certain confounding variables between the compared groups. Additionally, the median follow-up duration was relatively brief, slightly surpassing 3 years. Nevertheless, the study possesses undeniable strengths, including a substantial cohort derived from a tertiary referral center and the uniform evaluation of the therapy regimens administered to patients at the time of diagnosis. The treatment approach for all patients was based on consistent parameters, ensuring the homogeneity of both oncological and surgical interventions throughout the entire cohort.

We discovered that patients who underwent LC-CRT had better OS compared to those who received SCRT-delay. Additionally, an adverse MRI response is a sign of poorer outcomes for patients with LARC. The current neoadjuvant therapies do not seem to sufficiently improve the prognosis for the subgroup of patients with an unfavorable MRI response, underscoring the importance of developing new biological tools to identify these patients at the time of diagnosis.

## Data Availability

Data are not available due to legal restrictions.
